# Application of comprehensive u nit-based safety program model in the inter-hospital transfer of patients with critical diseases: a retrospective controlled study

**DOI:** 10.1186/s12913-021-06650-7

**Published:** 2021-07-13

**Authors:** Yimei Gu, Lina Liang, Liuna Ge, Ling Jiang, Xiaole Hu, Jing Xu, Yu Cao, Xiaoting Feng

**Affiliations:** Emergency intensive care unit (EICU), The First Affiliated Hospital of Anhui Medical University, Hefei, Anhui China

**Keywords:** Comprehensive unit-based safety program, Safety culture, Emergency intensive care unit, Inter-hospital transfer, Satisfaction

## Abstract

**Background:**

To explore the effect of applying a comprehensive unit-based safety program (CUSP) in the intrahospital transfer of patients with critical diseases.

**Methods:**

A total of 426 critically ill patients in the first affiliated Hospital of Anhui Medical University from August 2018 to February 2019 were divided into two groups according to the time of admission. Overall, 202 patients in the control group were treated with the routine transfer method, and 224 patients in the observational group were treated with the transfer method based on the CUSP model. The safety culture assessment data of medical staff, the occurrence rate of adverse events and related causes, the time of transfer, and the satisfaction of patients’ relatives to the transfer process were compared before and after implementation of the transfer model between the two groups.

**Results:**

Before and after the implementation of the CUSP mode transfer program, there were significant differences in the scores of all dimensions of the safety culture assessment of medical staff (*P* < 0.05), and the occurrence rate of adverse events and the causes in the observational group were significantly lower than those in the control group (disease-related, staff-related, equipment-related, environment-related) (*P* < 0.05). The transfer time for Computed Tomography (CT), Magnetic Resonance Imaging (MRI), operating room, and the interventional room was significantly shorter in the observational group than that in the control group (*P* < 0.05), while the satisfaction of relatives to the transfer process was significantly higher than those in the control group (*P* < 0.05).

**Conclusion:**

The implementation of CUSP model for the intrahospital transfer of critically ill patients can significantly shorten the in-hospital transfer time, improve the attitude of medical staff towards safety, reduce the occurrence rate of adverse events, and improve the satisfaction of patients’ relatives to the transfer process.

**Supplementary Information:**

The online version contains supplementary material available at 10.1186/s12913-021-06650-7.

## Background

Intrahospital transport (IHT) is the transfer between different departments of the same hospital [1]. The Emergency Intensive Care Unit (EICU) is for critically ill patients who are often transferred between EICU and other departments of the hospital for diagnosis or treatment interventions. In the current study, intrahospital transport mean patients going to Radiology Department, Catheter Laboratory, operating room, and other departments for examination and treatment. Knight et al. [2] found that during transport in the hospital, patients are at a risk for significant adverse events, such as airway/pulmonary complications, hemodynamic perturbations, nosocomial infections, acid/base disturbances, and glucose abnormalities. A retrospective cohort study of 1559 IHT by Veiga et al. [3] showed that the adverse events related to the clinical situations occurred in 117 (7.5%) transports, among which the incidence of hemodynamic instability was the highest (43). Non-clinical events occurred in 125 (8.0%) transports, while communication failures were the most frequent non-clinical events (99). The comprehensive unit-based safety program (CUSP) is a measure implemented at the unit (ward or institution) level to identify and eliminate patients safety problems and establish a safety culture within the unit [4]. This method was initially piloted at Johns Hopkins Hospital in 2001 [5]. In the medical field, CUSP aimed to improve the ability of teamwork and create a safety culture. The core of the method was to excavate the wisdom of front-line clinical staff, identify clinical problems, find the causes, and establish collaborative relationships with senior executives to resolve the issues, reduce risks, and improve the safety culture. Literature search retrieved a large number of studies on CUSP conducted worldwide; however, only a few studies had focused on the differences in cultural background. CUSP had been used in many medical institutions to improve the quality and safety of health care. For example, the program has been proven to be effective in reducing catheter-related urinary tract infections [6], surgical complications [7], nosocomial infection rates [8], and medication errors [9]. The application in the field of intensive care unit (ICU) was mainly focused on improving the management of mechanically ventilated patients [10] and reducing catheter-related blood flow infections [11]. None of the previous studies have introduced the CUSP program into the IHT transfer of critically ill patients. Thus, the present study introduced the CUSP program into the study of the IHT of critically ill patients in order to analyze the application effect of the method, the safety culture awareness of the medical and technical staff, the transfer time, the incidence and causes of adverse events, and the satisfaction of patients’ relatives with the transfer process that would form the basis of the clinical research.

## Methods

### Study participants

A total of 426 critically ill patients, from August 2018 to February 2019, were selected as the research subjects in the Department of Emergency Medicine, the first affiliated Hospital of Anhui Medical University. The patients were divided into two groups according to the time of admission. Among them, 202 critically ill patients from August to November 2018 comprised the control group that was treated with routine transfer methods, and 224 critically ill patients from December 2018 to February 2019 constituted the observational group that was treated with inter-hospital transfer based on the CUSP model. The inclusion criteria of the patients were as follows: (1) patients in EICU who needed IHT due to Computed Tomography (CT), Magnetic Resonance Imaging (MRI), interventional therapy, and operation; (2) age > 18-years-old; (3) signed informed consent form. However, since patients were in critical condition, all informed consents were signed by family members. Based on the exclusion criteria, those who were transferred from other Departments (Emergency, Ward, Operating room) to the EICU .

### Study design and implementation

This retrospective controlled study on the quality improvement and patient’s safety described the impact of the CUSP transfer program in our critically-ill cohort with respect to the safety culture assessment results of healthcare staff, factors related to adverse events due to the transfer, incidence and grade of adverse events, transfer time, and satisfaction of patients’ relatives to the transfer process.

CUSP mainly included the following processes: (1) The application process of CUSP model: ① downloading CUSP toolkit to understand the CUSP model; ② setting up a multidisciplinary team; ③ participation of administrative staff; ④ investigation of the current status of safety culture; ⑤ investigation of current status of adverse events in the IHT of critically ill patients. (2) Five steps of the CUSP model: ① safety knowledge training; ② safety risk; ③ administrative staff participation; ④ learning from defects; ⑤ communication and teamwork. (3) Follow-up work of CUSP model: ① assessment of safety culture status; ② safety quality assessment of IHT transfer of critically ill patients; ③ sharing the application effect of the CUSP model. All medical staff needed to be trained and qualified before they could participate in IHT of critically ill patients. The main contents included theoretical training and operation demonstration. The medical staff participated in objective structured assessment (theory test, cardiopulmonary resuscitation operation test, and simulation test for inter-hospital transfer of critically ill patients).

The control group received traditional IHT, and the observation group received CUSP mode. The measures of IHT in the two groups are shown in Table 1.

**Table 1:** Concrete measure of IHT of critically ill patients between control group and observational group

	control group (August 2018 to Novomber 2018) Traditional mode	observational group (December 2018 to February 2019) CUSP mode
Safety training	Three theoretical lectures, including the “Chinese guidelines for the transport of critically ill patients, 2010” (draft) [12]; maintenance of instruments and equipment related to transfer; emergency programs for sudden cardiac arrest, accidental extubation of artificial airway, failure of transfer ventilator, and insufficient oxygen source; demonstration of cardiopulmonary resuscitation operation and team first-aid during transfer. The nurses were required to work in the ICU for more than 1 year, the doctors needed to work in the ICU for > 6 months, participate in training, and pass the theory test, cardiopulmonary resuscitation test, and inter-hospital transfer simulation test for critically ill patients before they can independently carry out inter-hospital transfer for critically ill patients	① In addition to the traditional training methods, special safety inspection training modules, such as MRI safety instruction, enhanced CT safety instruction, and hyperbaric oxygen safety instruction, were added. ② Training needs attention with respect to the rescue vehicle and the use of drugs, and all the participating personnel is required to pass the examination. ③ Emphasis on the transfer model in the current situation, background, assessment, and recommendations (SBAR) [13]
Safety risks	clustered transfer list	optimizing and improving the clustered transfer list
Administrative staff participation	a special transfer elevator with a built-in telephone extension was set up in Building 1. b. Elevator workers go to work from 8:00 to 18:00	① Increasing transfer elevators (transfer-specific elevators were installed in each building). Eye-catching transfer elevator signs were pasted. Elevator workers wore chest card, and the service attitude was emphasized. ② Adding transfer goods and materials (adding 1 portable invasive ventilator, 2 portable monitors, and 1 transfer bed). ③ Unifying the items, drug types, quantity, and location of the materials in rescue vehicles of the whole hospital to facilitate the use of the second-level first aid site during the rescue. ④ Organizing safe transfer training in the hospital
Communication and cooperation	routine transfer	①Doctors should strengthen communication with the examination department and reduce the waiting time. ② Nurses should focus on health education and psychological nursing to appease the anxiety of the relatives. ③ Arranging for experienced medical technicians to perform the operation. ④ Reports of radiological examination should be obtained within 30 min. ⑤ SBAR model was used when patients were handover to the other departments
Learning from defects	quality control analysis of problems was performed every quarter, implementation of planning, implementation, inspection, adjustment, and improvement (plan, do, check, action, and PDCA) [14]	① Training medical staff to understand the root cause of the condition via lectures. ② Transfer defect analysis of critically ill patients was performed once a month, and simple root cause analysis was used to analyze the defects, including what happened, why, what should be done to reduce the risk, and how to confirm that the risk had been reduced

In addition to the above content, the observational group also established a standardized grading transfer process according to the characteristics of the critically ill patients and the actual situation of the clinical work. The specific content was as follows: (1) Evaluation and grading: it was the responsibility of the transfer decision-maker (main class and above physicians in the emergency room) to assess the patient’s condition (including vital signs, awareness, respiratory support, circulatory support, and major clinical problems) and expected transfer time to determine the transfer grade. The grading criteria were divided into grade I, II, and III according to the transfer risk from high to low, and the grade was determined according to the highest risk level corresponding to all the assessment items (for example: the patient’s vital sign, respiratory support was grade I, consciousness was grade III, and the patient’s transfer grade was determined as grade I). (2) Communication and explanation: a. communicating with the patient’s family to obtain informed consent, each IHT needs to be accompanied by the patient’s relatives; b. communicating with the receiving department to make the preparations accordingly. (3) Adequate preparation: includes the preparation of the transfer personnel, transfer equipment, and recipients. ① Transfer personnel preparation: the first was to select the corresponding medical staff in accordance with the requirements of the staffing standards for transfer classification; the second was to divide the transfer staff adequately and clarify their responsibilities. ② Transfer equipment preparation: one was to be equipped with the corresponding instruments and drugs in accordance with the requirements of the transfer grading equipment standard; the other was to debug and trial run the transfer instruments and equipment to find and resolve the issues promptly. ③ Patient preparation: evaluating the patient’s condition according to the transfer grade before departure, and carry out the transfer when the patient’s condition was stable, if possible. ④ Receiver preparation: informing the receiver of the patient’s condition and vital signs, the instruments and equipment used, the medication, and the time of arrival, in order to make the facility conducive to receive the patient. (4) Normal transfer: normal transfer should ensure the safety of the patients and medical personnel. ① In order to ensure the safety of the patients, healthcare workers must perform their respective duties and strive to complete the transfer work in the minimal duration. ② In order to ensure the safety of the healthcare personnel, transfer equipment should be placed in a standardized manner to avoid unnecessary accidents. (5) Response and management standardization: it primarily addresses the response and control of emergencies in the process of transfer. ① The aggravation of the patient’s condition should be treated by different transfer grade according to the following principles: the patients with grade I transfer should be rescued on the spot; the patients with grade II could continue to be transferred if their condition stabilized after preliminary treatment, otherwise they should return to the ward for rescue as soon as possible; the patients with grade III should return to the ward for treatment as soon as possible. ② For patients who cannot be examined and needed to wait, the general treatment principles were as follows: the allowable waiting time of patients with grade I should not exceed 5 min, that of patients with grade II should not exceed 10 min, and that of patients with grade III should not exceed 20 min. (6) Summary and evaluation: after the completion of the transfer, a comprehensive evaluation of the overall transfer work was carried out to provide a basis for the follow-up improvement of the transfer program and decision-making regarding the treatment of the patients. Moreover, the benefits and risks of patient transfer were evaluated, the stable condition, the rationality of the composition of transfer staff, the pertinence and predictability of planned measures, and the efficiency of communication were evaluated.

### Outcome measures

The transfer time, the assessment data of the safety culture of the medical staff, the analysis of factors related to adverse events, the incidence and grade of adverse events, and the satisfaction of patients’ relatives to the transfer process between the two groups before and after the application of the transfer model were compared.

Safety Attitude Questionnaire (SAQ) [15]: The safety culture of the medical staff in the emergency ICU was assessed using SAQ. A total of 4 surveys were conducted in August, November, December 2018 and February 2019, respectively. SAQ was a self-rating questionnaire, which was divided into six dimensions: job satisfaction, teamwork, working conditions, safety atmosphere, management perception, and stress perception. Likert 5 rating scale method (1 point indicated very disagree, 2 points indicated somewhat disagree; 3 points indicated neutral; 4 points indicated somewhat approbate, 5 points indicated very approbate) was used, with a total of 31 items. The higher the score, the more positive the safety attitude. The questionnaire was collected and checked on the spot, and the questionnaires with missing items > 3 were classified as invalid and excluded from the data analysis.

Criteria for the classification of transfer adverse events: According to the criteria proposed by Quenot et al. [16], the adverse events were divided into serious adverse events (such as cardiac arrest and re-intubation immediately after accidental extubation of the endotracheal tube), high-risk adverse events (such as increasing oxygen concentration or regulating ventilator parameters after the decrease of oxygen saturation, decreased blood pressure needed treatment, irritability of patients or man-machine antagonism, and pipe slippage), and events with hidden danger (mainly the events with the existence of hidden dangers, but no consequences). A registration book recorded the adverse events for inter-hospital transfer of critically ill patients.

Transfer time: It was the duration from when the patient was escorted out of his bed unit by medical staff until the return to the bed unit after examination or treatment was completed, which only included the time spent during the transfer and did not include the examination process, treatment process, and patient handover time (the handover was in accordance with the SBAR model specification). Some patients had multiple inter-hospital transfers (≥ 2) during the treatment and were treated with repeated registration in this study.

Satisfaction of patients’ relatives to the transfer process: The nurse in charge of the bed conducted a one-to-one face-to-face or telephone survey on the patient’s relatives within 48 h after the end of the inter-hospital transfer. A self-made satisfaction survey tool was used. It included 10 aspects: transfer staff, transfer process, transfer time, transfer goods, transfer route, transfer handover, medical cooperation, communication, and humanistic care. The full score was 100 points. Among them, 91–100 points indicated very satisfied, 81–90 points indicated satisfied, 71–80 points indicated general, 61–70 points indicated dissatisfied, < 60 points indicated very dissatisfied. Satisfaction = (satisfied + very satisfied) / total number of cases × 100%.

### Statistical analysis

IBM SPSS16.0 was used to analyze the data. The measurement data with normal distribution were expressed by mean ± standard deviation ($\overline{x}$ ± SD). The independent sample t-test was used for the comparison between the two groups of means. The enumeration data were expressed as a percentage, and the chi-square test was used for the comparison of the two rates or constituent ratios, and rank sum test was used for the comparison of the ranked data of multiple rates or constituent ratios. *P* < 0.05 was considered a significant difference.

## Results

### Baseline data of patients in the two groups

The observational group consisted of 122 males and 102 females, aged 24–78 (average: 60.64 ± 6.23)-years-old. A total of 112 patients did not use a ventilator, 26 patients used non-invasive ventilation, 89 patients received invasive ventilation, and 48 patients used vasoactive drugs. The control groups consisted of 102 males and 100 females, aged 28–76 (mean: 59.76 ± 6.48)-years-old. Of these, 98 patients did not use a ventilator, 23 patients were treated with non-invasive ventilation, 85 patients received invasive ventilation, and 45 patients used vasoactive drugs. However, no significant difference was detected in the general data between the two groups.

### Comparison of SAQ results between the two groups before and after the implementation of the model

The cohort comprised of 26 doctors, 78 nurses, and 8 medical technicians with a total of 112 subjects in the Emergency Department. The observational group sent out 112 questionnaires, including 108 valid questionnaires, and the effective rate was 96.43%, while the control group distributed 112 questionnaires, including 106 valid questionnaires, and the effective rate was 94.64%; no significant difference was detected in the effective rate between the two groups (*P* > 0.05). Before implementation, no significant difference in the SAQ results between the two groups of medical staff (*P* > 0.05). After implementation, the SAQ scores of each dimension of the medical staff in the observational group were significantly higher than those before implementation, the scores of SAQ of each dimension in the observational group were significantly higher than those in the control group, while the scores of SAQ of each dimension in the control group were not significantly different from those before implementation (*P* > 0.05) (Table 2).

**Table 2:** Comparison of SAQ results of medical staff of the two group (mean ± SD)

SAQ domain scores	Groups			
	Observational group (*n* = 108)		Control group (*n* = 106)	
	Before implementation	After implementation	Before implementation	After implementation
Job satisfaction	3.91 ± 0.26	4.27 ± 0.31^*#^	3.96 ± 0.31	4.05 ± 0.34
Teamwork climate	4.06 ± 0.34	4.36 ± 0.46^*#^	4.09 ± 0.36	4.15 ± 0.41
Working conditions	3.97 ± 0.27	4.32 ± 0.36^*#^	4.02 ± 0.31	4.15 ± 0.28
Stress recognition	4.15 ± 0.33	4.45 ± 0.41^*#^	4.11 ± 0.29	4.24 ± 0.35
Safety climate	3.43 ± 0.88	4.78 ± 1.31^*#^	3.54 ± 0.91	4.24 ± 1.12
Perception of management	3.96 ± 0.34	4.29 ± 0.36^*#^	4.03 ± 0.31	4.12 ± 0.34

Footnote:^*^compared to the same group before-implementation *P* < 0.05, ^#^compared to control group after-implementation *P* < 0.05

### Comparison of the occurrence rate and causes of adverse events between the observational and control groups

The occurrence rate of adverse events in the observational group was 18.30% (41/224), which was significantly lower than 37.62% (76/202) in the control group. The control group consisted of 12 cases of serious adverse events, 41 cases of high-risk adverse events, and 23 cases of events with hidden danger. The observational group comprised of 6 cases of serious adverse events, 30 cases of high-risk adverse events, and 5 cases of events with hidden danger. (Table 3). The grade of adverse events in the experimental and control groups was significantly lower than that in the control group, and a significant difference was noted between the two groups (*P* < 0.05). The analysis of the causes of adverse events showed that the occurrence rate of adverse events related to staff, equipment, and environment in the observational group was significantly lower than that in the control group (*P* < 0.05) (Table 4).

**Table 3:** Comparison of the incidence of adverse events in the two groups

adverse eventsn [n(%)]	Control group(*n* = 202)	Observational group(*n* = 224)	*χ*^*2*^***/***Z	*P*
Number of cases of adverse events	76 (37.62)	41 (18.30)	19.854	<0.001
Number of cases of adverse events	12 (5.94)	6 (2.68)	−3.007	0.003
High-risk adverse events	41 (20.30)	30 (13.40)		
Events with hidden danger	23 (11.39)	5 (2.23)

**Table 4:** Analysis of the causes of adverse events in the observational and control groups [n (%)]

causes of adverse events [n (%)]	Control group(n = 202)	Observational group(n = 224)	*χ*^*2*^	*P*
Disease-related	40 (19.80)	28 (12.5)	4.222	0.040
Staff-related	18 (8.91)	9 (4.02)	4.284	0.038
Equipment-related	6 (2.97)	0	4.779^#^	0.029
Environment-related	12 (5.94)	4 (1.79)	5.073	0.024

Footnote: ^#^4.77 was continuous correction

### Comparison of transfer time between the two groups

No significant difference was detected in the number of patients transferred between the two groups (*P* > 0.05). The transfer time in CT, MRI, operating room, and interventional room was significantly shorter in the observational group than that in the control group (*P* < 0.05) (Table 5).

**Table 5:** Comparison of transport time between the two groups of patients [n (%)]

Examination items	N/Time (min)	Group		*χ*^*2*^/*t*	*P*
		Observational group (*n* = 224)	Control group (*n* = 202)		
CT	n	203 (90.63)	189 (93.56)	1.246	0.264
	T	22.52 ± 4.41	27.54 ± 3.18	13.348	< 0.001
MRI	n	12 (5.36)	9 (4.46)	0.183	0.668
	T	34.43 ± 5.35	39.23 ± 5.92	8.79	< 0.001
Operation room	n	15 (6.70)	11 (5.45)	2.851	0.091
	T	16.44 ± 3.25	19.33 ± 3.69	8.594	< 0.001
Interventional room	n	12 (5.36)	10 (4.95)	2.719	0.099
	T	8.02 ± 2.33	11.53 ± 2.63	14.606	0

Footnote:Some patients had multiple examinations that need to be calculated repeatedly

### Comparison of the satisfaction of the patients’ relatives during the transfer process between the two groups

During the transfer process of the observational group, 32 cases of the patients’ relatives were very satisfied, 168 cases were satisfied, 21 cases were generally satisfied, 3 cases were dissatisfied, and 0 cases were very dissatisfied. The final overall satisfaction rate was 89.28% (200/224). During the transfer process of the control group, the relatives of the patients were very satisfied in 25 cases, satisfied in 140 cases, generally satisfied in 30 cases, dissatisfied in 8 cases, very dissatisfied in 0 cases, and the overall satisfaction rate was 81.68% (165/202). Strikingly, a significant difference was observed in the transfer satisfaction between the patients’ relatives in the two groups (Z = 4.992, *P* = 0.025).

## Discussion

Previous studies have shown that the occurrence rate of adverse events in the hospital was related to the safety culture of patients, and a positive safety culture reduces the occurrence rate of adverse events [17]. Beckmann et al. [18] pointed out that among the 900 factors related to adverse events of IHT of critically ill patients, system-related factors accounted for 46%, and personnel-related factors accounted for 54%, and both types were associated with the hospital safety culture. Adverse events of IHT of critically ill patients can be avoided or reduced by modifying the hospital safety culture. CUSP model promotes a safety culture and has been proven to be effective in many fields [19]. Therefore, it was necessary to apply the CUSP model to alter the hospital safety culture to reduce the adverse events of the IHT of critically ill patients.

The results of this study showed that the scores of SAQ of each dimension in the observational group were significantly higher than those in the control group, while the scores of SAQ of each dimension in the control group had no significant change as compared to those before implementation. The dimension of safety climate of control groups SAQ was positively improved at post-intervention (4.78 ± 1.31 in the intervention vs. 3.43 ± 0.88 in the control), indicating that CUSP training significantly improved the safety climate of ICU and the safety attitude of medical staff, in order to ensure the safety of patients. These phenomena were consistent with those in the study by Hsu et al. [20], wherein SAQ was used to investigate the ICU medical staff that implemented CUSP mode for 2 years, and the results showed that the dimension of the safety climate improved rapidly. A large-scale survey of 103 ICUs in the Michigan Keystone Project [21] confirmed that after multifaceted intervention by applying the CUSP model, the ICU medical staff had significantly improved with respect to teamwork, communication, and identifying and reducing risk. In another study, the safety culture of the surgical staff was improved. Hill et al. [22] pointed out thatthe training and management of the safety culture did not focus on completing every specific step but needed to connect each link together, starting from each link that might be risky; also, adaptive and corresponding changes were made that encouraged the members to find and raise problems. Based on the participation and emphasis, combined with the support of the leadership, the overall promotion of the safety culture and work, can be realized.

In a retrospective study of IHT in patients with acute myocardial infarction, Mueller et al. [23] pointed out that medical transfer often occurred in the hospital, which made patients face the potential risk of nursing interruption. Thus, patients and medical staff experienced some pressure. In a multicenter study in the ICUs in the USA, the positive awareness of the safety culture was related to the reduced occurrence rate of adverse events [24]. The results of this study showed that the occurrence rate of adverse events in the observational group was 18.30%, which was significantly lower than that in the control group (37.62%). The analysis of the reasons indicated that it might be related to the following aspects: Firstly, the CUSP model emphasized the importance of multidisciplinary teams and required stakeholders to participate and discuss the importance of inter-hospital transfer safety of critically ill patients, identify potential risks, and integrate these elements into a list for routine work. Secondly, patient safety was the responsibility of the whole medical system, and the observational group was allocated additional transfer equipment, and the transfer process was improved. Finally, the CUSP model involved medical, technical, and logistics administrators, who focused on the details of the transfer process, manpower and material resources invested in the program, and provided policy support. In the cause analysis, the occurrence rates of adverse events or hidden dangers caused by personnel-related, equipment-related, and environment-related events in the observational group were significantly lower than those in the control group. The analysis of the reasons indicated that these phenomena might be related to the following aspects. First, after the definition and classification of adverse events were determined in this study, the team discussed and studied, and the concept of medical and technical personnel changed. After realizing the importance of the safety of IHT of critically ill patients, the medical staff evaluated the patients carefully before the transfer, paid attention to the details, strengthened the evaluation of each link, and reduced the adverse events caused by the patient’s condition. Second, optimizing and using the cluster transfer list greatly reduced the situation of missing materials before transfer. In addition, after theoretical training, operation training, and objective structured examination, the staff was calm and skilled in the face of emergencies, could deal with the changes in the disease conditions appropriately and in a timely manner, and reduce the adverse events of transfer caused by personnel-related factors. Third, after each building was equipped with transfer elevators and full-time elevator workers, the patients did not have to wait for the elevator, which shortened the transfer time and avoided the lack of oxygen and the electricity of monitors and ventilators. Thus, the adverse events of transfer caused by environmental factors and equipment factors were also reduced.

The results of the study showed that the time spent in transferring patients to CT room, MRI, operating room, and interventional room in the observational group was 22.52 ± 4.41 min, 8.02 ± 2.31 min, 16.44 ± 3.25 min, 8.02 ± 2.3 min, respectively, which was significantly lower than that in the control group (27.54 ± 3.18 min, 39.23 ± 5.92 min, 19.33 ± 3.69 min, and 11.53 ± 2.63 min, respectively. Tabriz et al. showed that doctors’ participation in triage significantly reduces the patients’ waiting time for treatment [25]. The analysis of the reasons for the shortening of transfer time indicated that it might be related to the following aspects. First, after the setting up of special transfer elevators and full-time elevator workers, the waiting time for elevators during the transfer was reduced. Second, the optimized cluster transfer list was used to enable medical staff to prepare the supplies rapidly and reduce omissions. Third, communication with medical technology departments before the transfer was improved, which reduced unnecessary waiting. Fourth, the concept of medical and technical personnel had changed. The examination department arranged a technician with senior title, and the experienced technical personnel was skilled in the examination of critically ill patients, thereby shortening the duration of the process. After shortening the transfer time, not only the tension and anxiety of the patients and their relatives were alleviated, but also the time of the medical staff was saved. Also, insufficient oxygen storage caused by the long waiting time was reduced, and adverse events caused by insufficient electricity of monitors and ventilators were reduced. Therefore, shortening the transfer time had important clinical significance in reducing the adverse events of IHT of critically ill patients.

This study showed that positive safety culture not only promotes the patients’ safety [26] and increases satisfaction. The results of this study found that the satisfaction of the patients’ relatives in the observational group was 89.28%, while that in the control group was only 81.68%. The reasons were as follows: the improvement of the system, the addition of equipment, and the participation of multidisciplinary teams eliminated the hidden dangers for the transfer of critically ill patients and reduced the occurrence rate of adverse events. On the other hand, a positive safety culture [27] improved the safety and team environment in the medical unit. After the application of the CUSP model, doctors focused on the communication with the patients’ relatives, explaining the time, economics, and the physical cost while going to and from the hospital. Furthermore, the hospital made the overall arrangements, centralized the necessary examinations, and reduced the time spent on waiting for the examination. The nurses paid attention to health education and psychological nursing before the transfer, which relieved the anxiety and tension of patients. While arranging the examination of critically ill patients, medical technology departments provided senior medical and technical personnel as possible. These parameters rendered the technique and communication effective, which made the patients and their relatives feel safe.

## Conclusions

Herein, the CUSP program was introduced into the study of hospital transfer of critically ill patients, which optimized the preparation and process at each stage of transfer while improving the attitude of medical staff towards safety culture. The results showed that the effect was obvious in the occurrence rate of adverse events, transfer time, and satisfaction of patients’ relatives to the transfer process. It had a significant clinical application in promoting patients’ safety. Nevertheless, the present study also had some limitations: (1) the sample size was small, and the research duration was relatively short; (2) because of the time constraint, only quantitative research was conducted in this study. Thus, it was suggested to expand the sample size in the future studies and combine the quantitative and qualitative research to further understand the degree and inner thoughts of medical staff in the application of the CUSP model; (3) This study failed to track and evaluate the long-term effect of inter-hospital transfer program based on the CUSP model. Additional studies should further expand the sample size and develop large-sample, multicenter randomized controlled trials to track the effect of the intervention for a prolonged period, in order to better evaluate the effectiveness of the intervention program. It can also be used to resolve the safety concerns of the patients, such as reducing nosocomial infection, accidental extubation rate, fall incidence, and pressure sore incidence.

## Supplementary Information

**Additional file 1.**

## Data Availability

The datasets used and analyzed during the current study are available from the corresponding author.

## Abbreviations

CUSP: Comprehensive unit-based safety program
CT: Computed tomography
MRI: Magnetic resonance imaging
IHT: Inter-hospital transfer
EICU: Emergency intensive care unit
SBAR: Situation background assessment recommendation
SAQ: Safety attitude questionnaire
