# Supplementary material for: Application of comprehensive u nit-based safety program model in the inter-hospital transfer of patients with critical diseases: a retrospective controlled study
Source: BMC Health Serv Res. 2021 Jul 13;21:690. doi: 10.1186/s12913-021-06650-7 (PMC8275901; doi:10.1186/s12913-021-06650-7)
Supplement: Supplementary file 1 — Additional file 1. [file 12913_2021_6650_MOESM1_ESM.doc]

Dear editors

We had replied reviewers reports.

Re: Manuscript reference No BHSR-D-20-02630

Please find attached a revised version of our manuscript “BHSR-D-20-02630 based Manuscript modified version”.

Your comments and those of the reviewers were highly insightful and enabled us to greatly improve the quality of our manuscript.Revisions in the text are shown using yellow highlight for additions, and strikethrough font [~~example~~] fordeletions. Regarding the second modification, the corresponding part of the text has been modified (the font has been marked in red) for the supplement of

the formatted content.In the following pages areour point-by-point responses to each of thecomments of the reviewers as well as your own comments.

Responses to the comments of Reviewer #1

1- The paper should be edited by a native English speaker.

Answer：The paper had edited by a native English speaker.

2- The way the information presented should be modified.For example, many

results are introduced in the discussion,limitations are discussed in the conclusion.

Answer：we had introduced many results in the discussion,discussed limitations in the conclusion.

3- At the discussion section, the authors may cite previous related studies such as Tabriz AA, Trogdon JG, Fried BJ.Association between adopting emergency department crowding interventions and emergency departments' core performance measures. The American Journal of Emergency Medicine. 2020 Feb 1;38(2):258-65. In this paper, the authors showed interventions such as the one you

talked about significantly improve outcomes.

**Answer：**Thank you for your good suggestions. After reading, this paper will be used as a reference for this study 25.on P27，line608-611.

4- In tables, why the P-value presented in the row instead of columns?

Answer：We have made adjustments，P-value presented in the row.Table 3、Table 4.

Responses to the comments of Reviewer #2

1-Title

It is not clear in the title or in the manuscript how the authors defifine inter-hospital transfers and what transports the included patients went through. Was in inside the hospital (intra-hospital transports) or between hospitals (inter-hospital transfers). This needs to be clarifified throughout the manuscript

Answer：It is intra-hospital transports，we have defined it. On p 4，line 53-56.

2-Background

In the fifirst paragraph, the authors refer to studies (ref 1-3) without describing the context of these studies/ research. Please elaborate the text.

Answer：We were edited this paragraph and explained the research background of the inferences.on p 4，line 56-63.

3-Methods

Research subjects

On p 4, line 97 you state that an inclusion criterion was signed informed consent form. In the results (p 12) you report that many patients were on ventilator care. When and how did the patients in the different groups sign the form? Did you have any dropouts because of the patients' ability to give their consent?

Answer：As the patients in EICU were critically ill Patients, all informed consents were signed by their families.on p6，line107-108.EICU patients for more intensive patients, can't cooperate with signature, etc., considering the intervention does not involve the treatment, so will not cause harm to the patient's autonomy, in addition, based on the cultural background in our country, for the protection of patients with mental health, reduce unnecessary psychological burden we choose by patients' families signed informed consent form and participate

in the study.

4-Study design and implementation

This part of the manuscript is very extensive, contains many enumerations and encompasses fifive pages of the manuscript. To make it more readable I suggest the authors to rework this part. This could be done by presenting the CUSP-model as well as details about the different groups in tables or textboxes.

Answer：we had reworked this part，presented the CUSP-model as well as details about the different groups in Tables 1.

5-P 5 Line 111, You write: CUSP was widely used in the fifield of health care. Consider the use of tempus and add appropriate references

Answer：Considering the strictness of this manuscript, we deleted this sentence.on P6，line118.

6-Involved health care personnel. Was it the same personnel who carried out transfers of both patient groups, for me this is not clear.

Answer：it was the same personnel who carried out transfers of both patient groups,on P14，line302-306.

7-When was the staff training for the "control group" and the "observational group" carried out?

Answer：the staff training for the "control group" carried out from August 2018 to Novomber 2018,and the "observational group" carried out from December 2018 TO February 2019,**table 1**.

8-Evaluation variables

Relatives´ satisfaction: This evaluation was performed by telephone after the transfer. It is somewhat unclear what items the survey covered. How could relatives evaluate the transfer process, did they accompany their loved ones during the transfers?

Answer：Using self-made survey tools, including 10 aspects, transfer personnel, transfer process, transfer time, transfer goods, transfer route, transfer handover, medical cooperation, communication and humanistic care,relatives had completed

all items,on P13，line272-274.

9-Results

SAQ: From the headline and table 2, it appears the two groups consisted of different people. In the text a cohort comprising 112 subjects is presented, please explain.

Answer：I'm sorry that there is an error in the expression here. In fact, the SAQ scores of the same group of medical staff were compared before and after the intervention. Based on this, we modified the title of Table 2.

10-Discussion

The discussion follows the results and seems adequate, but the way of using references in the discussion is somewhat confusing. For example, on p. 16 (line 371-272) reference 21 and 22, it is unclear if the text belongs to the actual study or the to the references results.

Answer：we had clarified these sentences,on P18，line394-399.

11-P 18, line 431. The authors write that the study improved patients' experience of hospitalization. I cannot find how this is investigated or presented in the manuscript. In addition, in this paragraph the unclear way of using references (23-25) is repeated.

Answer：Considering the strictness of this manuscript, we deleted this sentence that the study improved patients' experience of hospitalization.on P20，line460.we had used references correctly.

**An addition to the formatted content**

**Question1-** Author's contributions:

Please represent authors' names using their full initials, not their full name, in the Authors’ Contributions section. For example, the initials of John Smith would be ‘JS’. If there are any duplicated initials, please differentiate them to make it clear that the initials refer to separate authors, for example, by adding their middle name initial.

Answer：According to your good suggestions, we have modified the corresponding part and marked it in red. See details for details P23，line520-524.

**Question2-**Title page:

Please include a title page as the fifirst page of your manuscript where you

list the title of the manuscript. The corresponding author and his/her email address should be indicated

Answer：We have modified this part of content,it's marked at the bottom of

the P1.

**Question3-** Declarations

Please add the heading "Declarations" before you list the Declarations subheadings. In your manuscript, this will be directly before the “Ethics and Consent

to participate” heading.

Answer:We have responded to this question before the headline, on P1,line1.

**Question4-** Please complete and reformat the list of sub-section format

as seen below:

Ethics approval and consent to participate

Consent for Publication

Availability of data and material

Competing interests

Funding

Authors' contributions

Acknowledgements

Answer：We have reorganized the above content sequence according to your suggestion,on P20,line507-529,as follows:

**Ethics approval and consent to participate**

The study was approved by the Medical Ethics Committee of the Anhui Medical University. Written informed consent was obtained from all the patients’ relatives.

**Consent for Publication**

Not Applicable.

**Availability of data and material**

The datasets used and analyzed during the current study are available from the corresponding author.

**Competing interests**

The authors declare that they have no competing interests.

**Funding**

None..

**Authors' contributions**

GYM and LLN carried out the studies, participated in collecting data, and drafted the manuscript. GLN and JL performed the statistical analysis and participated in its design. HXL, XJ, CY, and FXT helped to draft the manuscript. All authors read and approved the final manuscript.

**Acknowledgements**

This study was supported by the Emergency intensive care unit (EICU), The First Affiliated Hospital of Anhui Medical University. We appreciated the critically ill patients and their relatives for their willingness to participate and cooperate with the present study. The authors confirmed that there was no conflict of interest.

**Author details**

All the authors were from the Emergency Intensive Care Unit of the First Affiliated Hospital of Anhui Medical University,Hefei Anhui, China

Your and the reviewers’ comments have again stimulated changes we feel further improved the paper. Should you find the paper requiresfurther clarification or revision, we most certainly stand ready to do so.We would be happy if the manuscript will be evaluated by your Editorial Board members for publication in **BMC HEALTH SERVICES RESEARCH*.***

We shall look forward to hearing from you at your earliest convenience.

With best regards,

Yours sincerely,

Lina Liang

Emergency intensive care unit (EICU), The First Affiliated Hospital of Anhui Medical University, Hefei Anhui, China

Email: 305851731@qq.com
